# Dietary nucleotides supplementation during the suckling period improves the antioxidative ability of neonates with intrauterine growth retardation when using a pig model†

**DOI:** 10.1039/c8ra00701b

**Published:** 2018-04-30

**Authors:** Liang Hu, Xie Peng, Linlin Qin, Ru Wang, Zhengfeng Fang, Yan Lin, Shengyu Xu, Bin Feng, De Wu, Lianqiang Che

**Affiliations:** Institute of Animal Nutrition, Sichuan Agricultural University No. 211, Huimin Road, Wenjiang District Chengdu 611130 Sichuan People's Republic of China clianqiang@hotmail.com +86-835-2883166 +86-835-2882828; Key Laboratory for Animal Disease-Resistance Nutrition of China Ministry of Education Chengdu 611130 Sichuan People's Republic of China

## Abstract

The aim of the present study was to investigate the effect of dietary nucleotides supplementation on the antioxidant status of piglets affected by intrauterine growth retardation (IUGR). Fourteen pairs of normal birth weight (NBW) and IUGR piglets were fed either a control diet (CON) or a nucleotides supplementation diet (NT) from 7 d of age to 28 d postnatal. Blood, liver and jejunum samples were collected at the end of the study. The results showed that IUGR piglets had decreased (*P* < 0.05) concentrations of plasma total antioxidant capability (T-AOC) and total superoxide dismutase (T-SOD), gene expressions of hepatic cytoplasmic copper/zinc SOD (CuZnSOD) and PPARγ coactivator-1α (PGC-1α) and jejunal glutathione peroxidase (GP_X_) and extracellular superoxide dismutase (ESOD), accordingly, there was markedly higher (*P* < 0.05) plasma malondialdehyde (MDA) and hepatic and jejunal mitochondria DNA content in the IUGR piglets relative to NBW piglets. Regardless of body weight, dietary NT supplementation significantly increased (*P* < 0.05) plasma concentrations of T-AOC, T-SOD, CuZnSOD, GP_X_ and the ratio of reduced glutathione to oxidized glutathione, hepatic T-SOD, GP_X_ and mitochondria DNA content, while hepatic MDA concentration was markedly decreased (*P* < 0.05) 19.1% by NT diet. Furthermore, the gene expressions of hepatic glutathione reductase, CuZnSOD, nuclear erythroid 2-related factor 2, PGC-1α and nuclear respiratory factor-1 (NRF-1) and jejunal GP_X_, CuZnSOD, ESOD and NRF-1 were significantly increased (*P* < 0.05) by NT diet, whereas the gene expression of Kelch-like ECH-associated protein 1 were markedly decreased (*P* < 0.05) compared with that of piglets fed with CON diet. These results indicate that dietary NT supplementation prevents the effect of IUGR on oxidative status and mitochondria DNA damage through improving the non-enzymatic and enzymatic antioxidant capacities as well as mitochondria biogenesis of piglets.

## Abstract 2

The aim of the present study was to investigate the effect of dietary nucleotides supplementation on the antioxidant status of piglets affected by intrauterine growth retardation (IUGR).

## Graphical abstract

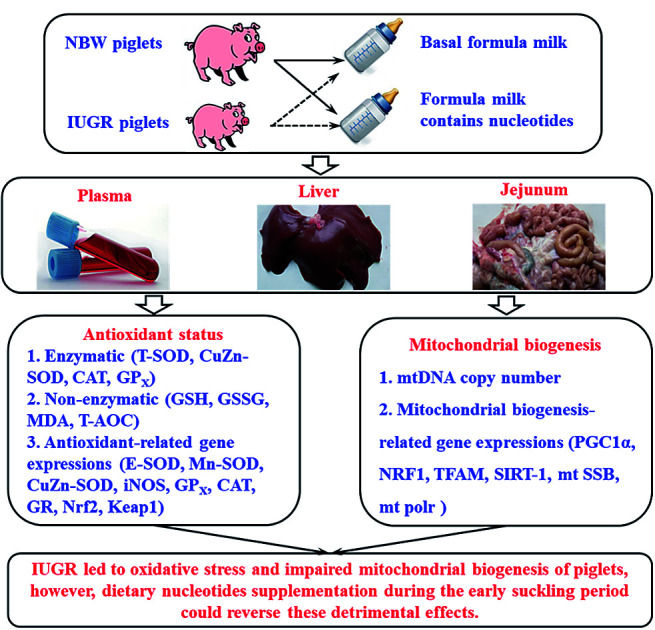

## Introduction

The concept of “fetal programming” suggested that the inadequate uptake of nutrients during intrauterine development leads to abnormal growth of the fetus and causes a long-term influence on the offspring.^1^ Intrauterine growth retardation (IUGR) is defined as impaired growth and development of the embryo and/or its organs during gestation.^2^ In human beings, approximately 5–10% of infants worldwide suffer from IUGR.^3^ There is growing evidence that neonates with IUGR suffer serious oxidative stress after birth, as evidenced by increased reactive oxygen species (ROS) generation and oxidative damage.^4–6^ Furthermore, in order to achieve rapid growth, neonates with IUGR are generally fed a highly nutritional diet (such as, high fat diet), which aggravates oxidative stress contributing to a high risk of metabolic diseases in adult life.^7,8^ Oxidative stress also plays an important role in signal transduction and transcription of specific genes through regulating mitochondrial biogenesis,^9^ the increased oxidative stress may contribute to alterations in the abundance of mitochondria as well as the copy number and integrity of mitochondria DNA.^10^

Nucleotides, as the building blocks of nucleic acids, are a group of bioactive agents playing important roles in nearly all biochemical processes, such as transferring chemical energy, biosynthetic pathways and coenzyme components.^11,12^ The nucleotides requirement could be met by three sources including *de novo* synthesis, salvage pathways and food. Generally the milk of mammal animals have higher contents than any other food origin.^13^ It has been demonstrated that nucleotides supplementation had positive effects on small-intestinal growth and development, stimulation of systemic immunity and lipid metabolism.^14,15^ Moreover, nucleotides supplementation effectively eliminates the DNA damage under oxidative stress in pigs and chickens.^16,17^ Dietary nucleotides supplementation could also improve health status and extend the life span through enhancing antioxidant capability.^18,19^ However, little is known on the effects of dietary nucleotides supplementation on non-enzymatic and enzymatic antioxidant systems of the plasma and organs in neonates with IUGR. Due to the physiological and genomic similarities between pigs and humans, pigs have been recognized as an ideal model for the study of clinical nutrition.^20^

The purpose of the current study was conducted to investigate whether dietary nucleotides supplementation can favorably affect oxidative status of IUGR piglets. The finding of the current study may provide new way to alleviate oxidative damage resulted from in utero maldevelopment.

## Materials and methods

The study protocol was reviewed and approved by the Care and Use Committee of Sichuan Agricultural University, and followed the current laws of animal protection (Ethic Approval Code: SCAUAC201408-3).

### Animals, experimental design, and formula milk

The study was carried out on a subgroup of animals from a previous study, and all details of the experimental protocol have been described.^21^ Briefly, fourteen pairs of normal birth weight (NBW) male piglets (Pig Improvement Company 327 × 1050) at 1.56 (SD 0.05) kg and IUGR littermates at 0.91 (SD 0.03) kg were selected from the 14 healthy pregnant sows, who had same litter size (10 live piglets per litter). The criteria of choosing piglets was based on our previous study.^22,23^ All piglets were weaned at 7 day of age and moved to be individually fed with milk-based diet every 3 hours by bottle feeding between 06.00 and 24.00 hours in nursing cages (0.8 m × 0.7 m × 0.4 m). For nutritional treatments, 7 pairs of NBW and IUGR piglets were assigned to receive control diet (CON), while the other 7 pairs were allocated to receive nucleotides-supplemented diet (NT). Therefore, four groups of piglets were created and studied: NBW-CON; IUGR-CON; NBW-NT; IUGR-NT (*n* = 7). Milk replacer powder was formulated according to the previous study in our lab and the composition was shown in ESI Table 1.†^24^ The milk-based diet was prepared by mixing 1 kg of formula powder with 4 litres of water to a milk solution. The nucleotide-supplemented diet was prepared by adding milk replacer powder with a mixture of pure nucleotides (29.6 g 5′-adenine-monophosphate, 14.2 g 5′-cytosine-monophosphate, 40.8 g 5′-guanosine-monophosphate, 5.8 g 5′-inosine-monophosphate, and 650.5 g 5′-uridine-monophosphate), a total of 740.9 g nucleotides per 100 kg milk replacer powder, which according to the average content of nucleotides in sow milk during day 7 to day 28 after birth.^25^ Pure nucleotides were donated by Zhen-AO Group Co. Ltd. (Dalian, China) and had purities of 97%, according to the analysis of the manufacturer. All piglets had free access to drinking water. Room temperature was maintained at approximately 30 °C and the humidity was controlled between 50% and 60%. This trial lasted 21 days.

### Sample collection

Blood samples were collected by venepuncture on the morning (08.00 hours) of day 21 after an overnight fast, a 10 mL blood sample was collected by venipuncture of jugular vein. Blood samples were injected into vacuum tubes containing sodium heparin and allowed to coagulate for 40 min before centrifugation (10 min, 2375 × *g* at 4 °C), the plasma samples were stored at −80 °C until analysis. After blood sampling, all piglets were anaesthetized with an intravenous injection of pentobarbital sodium (50 mg kg^−1^ BW) and slaughtered. The liver and jejunal samples were snap frozen and stored at fridge with −80 °C until further analysis.

### Measurement of the antioxidant parameters and lipid peroxidation in the plasma and liver

The clotted plasma sample was centrifuged at 2000 × *g* at 4 °C for 20 min before assays. Liver sample was defrosted and homogenized on ice with 10 volumes of cold buffer consisting of 250 mmol L^−1^ sucrose, 5 mmol L^−1^ Tris–HCl, and 0.1 mmol L^−1^ edetic acid–2Na (pH 7.5). The homogenate was centrifuged at 4000 × *g* at 4 °C for 15 min to obtain the supernatant for biochemical assays. The plasma and homogenate were used to determine the content of malondialdehyde (MDA), oxidized glutathione (GSSG), reduced glutathione (GSH), enzymatic activity of catalase (CAT), total superoxide dismutase (T-SOD), CuZn superoxide dismutase (CuZn-SOD), glutathione peroxidase (GP_X_) and total antioxidative capability (T-AOC) using commercial assay kits (Nanjing Jiancheng Institute, Jiangsu, China). Protein concentration of liver tissue was determined using bicinchoninic acid (BCA) as a detection reagent for Cu^+^ following the reduction of Cu^2+^ by protein in an alkaline environment (BCA protein kit, Sangon company, China). All samples were measured in duplicate.

### Assay of T-AOC in the plasma and liver

Because the antioxidant defense system consists of enzymatic and non-enzymatic antioxidants that reduce Fe^3+^ to Fe^2+^, in this study T-AOC was measured by the reaction of phenanthroline and Fe^2+^ using a spectrophotometer (Beckman DU-800, Los Angeles, CA, USA) at 550 nm. In this assay, 1 unit (U) represents the 0.01 increase in the absorbance value in 1 min per milligram of tissue protein or per milliliter of plasma. The T-AOC activity was presented as U mL^−1^ (in plasma) or U mg^−1^ protein (in tissue).

### Assay of MDA in the plasma and liver

MDA were quantified using the thiobarbituric acid (TBA) method according to Hou *et al.*^26^ Briefly, MDA content was assayed by reacting with TBA in acidic medium for 30 min at 95 °C to generate a resultant pink product that can be spectrophotometrically determined at 532 nm. The content of MDA was presented as nmol mL^−1^ (in plasma) or nmol mg^−1^ protein (in tissue).

### Assay of GSH and GSSG in the plasma and liver

The GSH and GSSG were estimated by the procedure of Moron *et al.*^27^ Briefly, total glutathione content was determined by calculating the rate of reduction of 5,5′-dithiobis-2-nitrobenzoic acid by GSH at 412 nm and comparing this with a GSH standard curve. GSSG content in the samples was determined using the same method after treating the samples with 4-vinylpyridine for 60 min. GSH content was calculated as follows: total glutathione content − 2 × GSSG content. The GSH and GSSG were presented as μmol L^−1^ (in plasma) or nmol mg^−1^ protein (in tissue).

### Assay of SOD in the plasma and liver

The activity of SOD was measured spectrophotometrically at 550 nm using a method described Jia *et al.*^28^ This technique involves the decrease of the product (superoxide ions) of the xanthine/xanthine oxidase system and the formation of red formazan by reacting with 2-(4-iodophenyl)3-(4-nitrophenol)-5-phenyltetrazolium chloride. SOD activity was presented as U mL^−1^ (in plasma) or U mg^−1^ protein (in tissue), and 1 U of SOD denotes 50% of inhibition of superoxide ion production in the reaction.

### Assay of GP_X_ in the plasma and liver

The activity of GP_X_ was determined by quantifying the rate of H_2_O_2_-induced oxidation of GSH to GSSG as described previously.^29^ One unit of GP_X_ was defined as the amount required to reduce the level of GSH by 1 mmol mL^−1^ in 5 min per 0.1 mL of plasma or homogenate. The activity of GP_X_ was expressed as U mL^−1^ (in plasma) or U mg^−1^ protein (in tissue).

### Assay of CAT in the plasma and liver

The activity of CAT was measured by the methods of Özmen *et al.*^30^ The enzymatic reaction is terminated by the addition of ammonium molybdate, which generates a light-yellow composite that can be measured at 405 nm. CAT activity was expressed as U mL^−1^ (in plasma) or U mg^−1^ protein (in tissue), and 1 U of CAT is defined as the amount of enzyme needed to decrease 1 mmol L^−1^ of H_2_O_2_ at the 37 °C and for 1 s per milligram of tissue protein or per milliliter of plasma.

### Total RNA extraction

Total RNA was extracted from the frozen liver and jejunum tissues (approximately 100 mg) using Trizol Reagent (Invitrogen, Carlsbad, CA, USA), according to the manufacturer's instructions. RNA integrity and quality were determined by agarose gel electrophoresis (1%) and spectrometry (A260/A280). RNA concentration was confirmed by nucleic-acid/protein analyzer (Beckman DU-800, CA, USA). After determining the RNA concentration, 1 μg of total RNA was reverse-transcribed into complementary DNA (cDNA) using a PrimeScripte RT Reagent Kit (TaKaRa Biotechnology, Dalian, Liaoning, China) following manufacturers' instructions. The RT products (cDNA) were stored at −20 °C for relative quantification by polymerase chain reaction (PCR).

### Real-time PCR analysis

Primers were designed by Primer Express 3.0 (Applied Biosystems, Foster City, CA, USA) and shown in ESI Table 2.† cDNA was amplified using a real-time PCR system (ABI 7900HT, Applied Biosystems, USA). The mixture (10 μL) contained 5 μL of SYBR Green Supermix (TaKaRa, Japan), 1 μL of cDNA, 0.4 μL of each primer (10 μM), 0.2 μL of ROX reference dye and 3 μL of ddH_2_O. The cycling conditions were used as follows: denaturation at 95 °C for 15 s, followed by 40 cycles of denaturation at 95 °C for 5 s, annealing at 60 °C for 30 s, and extension step at 72 °C for 15 s. Product size was determined by agarose gel electrophoresis. The standard curve of each gene was run in duplicate and three times for obtaining reliable amplification efficiency values as described previously.^31^ The correlation coefficients (*r*) of all the standard curves were >0.99 and amplification efficiency values were between 90 and 110%. The most stable housekeeping genes (β-actin and GADPH) were chosen for normalization. Relative mRNA abundance was determined using the Δ cycle threshold (Δ*C*_t_) method, as outlined in the protocol of Applied Biosystems. In brief, a Δ*C*_t_ value is the *C*_t_ difference between the target gene and the reference gene (Δ*C*_t_ = *C*^target^_t_ − *C*^reference^_t_). For each of the target genes, the ΔΔ*C*_t_ values of all the samples were calculated by subtracting the average Δ*C*_t_ value of the corresponding IUGR-CON group. The ΔΔ*C*_t_ values were converted to fold differences by raising 2 to the power −ΔΔ*C*_t_ (2^−ΔΔ*C*_t_^) according to Livak and Schmittgen (2001).^32^

### Analysis of mitochondrial DNA content

Total DNA was extracted from the liver and jejunum of each piglet using a DNAiso reagent (DP304-02, TIANGEN BIOTECH, China). All the procedures were according to the manufacturer's protocol. The content of mitochondrial DNA (mtDNA) relative to nuclear genomic DNA was measured by coamplifying the Mitochondrial D-loop (mt D-loop) and the nuclear-encoded β-actin gene using real-time PCR assay. The amount of mt D-loop and β-actin gene were quantified by fluorescent probes. The sequence of primers and probes were shown in ESI Table 3.† PCR amplification was carried out in a 20 μL reaction volume consisting of 8 μL TaqMan Universal Master Mix, 1 μL enhance solution, 1 μL each of forward and reverse primers, 1 μL probes, 7 μL ddH_2_O and 1 μL DNA. The cycling conditions were as follows, 95 °C for 10 s, 50 cycles involving a combination of 95 °C for 5 s and 60 °C for 25 s, and 95 °C for 10 s. Each sample was amplified in triplicate. The fluorescence spectra were monitored by Real-Time PCR detection System (ABI 7900HT, Applied Biosystems, USA). The ratio of mtDNA to genomic DNA content was calculated using the method of 2^−ΔΔ*C*_t_^, as described previously.^32^

### Statistical analysis

Results are presented as means with their standard errors. Data were analysed using the MIXED procedure of Statistical Product and Service Solutions 20.0 (Chicago, IL, USA) according to the following model: *y*_*ijk*_ = *μ* + *a*_*i*_ + *b*_*j*_ + (*ab*)_*ij*_ + *e*_*ijk*_ (*i* = 1, 2, *j* = 1, 2, *k* = 1, 2, … ,14), where *y*_*ijk*_ represents the dependent variable, *μ* is the mean, *a*_*i*_ is the effect of BW (IUGR, NBW), *b*_*j*_ is the effect of diet (CON, NT), (*ab*)_*ij*_ is the interaction between BW and diet, and *e*_*ijk*_ the error term. The normality and homogeneity of variances were evaluated by Shapiro–Wilk *W*-test and Levene's test respectively. Duncan's multiple range test was used for multiple comparison *post hoc* when the effects of BW, diet or their interaction was significant. Probability values <0.05 were considered statistically significant, and values between 0.05 and 0.10 were considered to indicate trends.

## Results

### Antioxidant-related enzyme activities in plasma

As shown in Table 1, regardless of diet, plasma T-SOD (by 8.6%) and T-AOC (by 14.8%) concentration of IUGR piglets was lower than that of NBW piglets (*P* < 0.05), while plasma MDA concentration (by 39.3%) in IUGR piglets was higher than that of NBW piglets (*P* < 0.05). Moreover, IUGR had a tendency to decrease the concentration of GP_X_ (by 9.8%) in the plasma of piglets (*P* = 0.078). Regardless of BW, dietary NT supplementation significantly increased the content of T-AOC (by 19.6%), T-SOD (by 6.5%), CuZn-SOD (by 11.1%), GP_X_ (by 12.4%) and the ratio GSH to GSSG (by 17.7%) in the plasma of piglets (*P* < 0.05). Additionally, the concentration of plasma GSH (by 19.3%) had a tendency to be increased by dietary NT supplementation (*P* = 0.075).

**Table 1:** Effect of dietary nucleotides supplementation on systematic oxidative status of intrauterine growth retarded (IUGR) and normal birth weight (NBW) neonates

Items	CON		NT		SEM	*P* values		
	NBW	IUGR	NBW	IUGR		BW	Diet	BW × diet
T-AOC (U mL^−1^)	2.13^b^	1.64^a^	2.34^b^	2.17^b^	0.09	0.048	0.030	0.319
CAT (U mL^−1^)	11.49	10.27	11.75	11.28	0.63	0.524	0.630	0.774
T-SOD (U mL^−1^)	40.35^b^	35.48^a^	41.46^b^	39.33^b^	0.63	0.001	0.018	0.173
CuZn-SOD (U mL^−1^)	25.85^a,b^	24.17^a^	27.98^b^	27.60^b^	0.50	0.247	0.004	0.456
MDA (nmol mL^−1^)	5.12^a^	7.49^b^	4.84^a^	6.38^a,b^	0.36	0.005	0.272	0.513
GSSG (μmol L^−1^)	2.66	3.24	2.94	2.87	0.12	0.283	0.856	0.174
GSH (μmol L^−1^)	9.19	9.63	11.48	10.97	0.50	0.971	0.075	0.627
GSH/GSSG	3.47^a,b^	3.08^a^	3.90^b^	3.81^a,b^	0.13	0.338	0.027	0.539
GP_X_ (U mL^−1^)	333.27^a,b^	296.84^a^	370.36^b^	337.86^a,b^	10.14	0.078	0.048	0.917

### Antioxidant-related enzyme activities in the liver

As shown in Table 2, irrespective of diet, IUGR had a tendency to decrease the concentration of T-SOD (by 6.1%, *P* = 0.062) and the ratio of GSH to GSSG (by 10.9%, *P* = 0.100) in the liver of piglets. Regardless of BW, dietary supplementation NT significantly increased the concentrations of T-SOD (by 7.8%, *P* < 0.05) and GP_X_ (by 11.4%, *P* < 0.05), whereas markedly decreased the concentration of MDA in the liver of piglets (by 19.1%, *P* < 0.05). Furthermore, BW and diet had a significant interaction effect on the ratio of GSH to GSSG in the liver of piglets (*P* < 0.05).

**Table 2:** Effect of dietary nucleotides supplementation on liver oxidative status of intrauterine growth retarded (IUGR) and normal birth weight (NBW) neonates

Items	CON		NT		SEM	*P* values		
	NBW	IUGR	NBW	IUGR		BW	Diet	BW × diet
T-AOC (U mg^−1^ protein)	2.12	2.01	3.24	2.25	0.09	0.311	0.086	0.429
CAT (U mg^−1^ protein)	51.55	44.72	51.70	49.65	2.75	0.524	0.630	0.774
T-SOD (U mg^−1^ protein)	56.15^b^	50.68^a^	58.32^b^	56.86^b^	1.01	0.062	0.027	0.269
CuZn-SOD (U mg^−1^ protein)	36.20	33.84	35.14	37.41	0.66	0.976	0.341	0.087
MDA (nmol mg^−1^ protein)	9.70^a,b^	10.78^b^	7.91^a^	8.65^a^	0.36	0.155	0.004	0.784
GSSG (nmol mg^−1^ protein)	8.27	9.29	8.57	8.17	0.29	0.612	0.502	0.248
GSH (nmol mg^−1^ protein)	54.27	47.55	54.16	53.86	1.44	0.228	0.286	0.269
GSH/GSSG	6.86^b^	5.19^a^	6.40^a,b^	6.62^b^	0.23	0.100	0.259	0.035
GP_X_ (U mg^−1^ protein)	81.19^a,b^	75.05^a^	90.45^b^	83.58^a,b^	2.21	0.127	0.041	0.931

### Antioxidant-related genes in the liver

Table 3 presents the mRNA levels of antioxidant-related genes in the liver. IUGR piglets had significantly decreased the mRNA expression of CuZn-SOD (by 20.3%) in the liver relative to NBW piglets (*P* < 0.05). Moreover, IUGR had a tendency to decrease the mRNA expression of GP_X_ (by 17.4%, *P* = 0.067) and Nrf2 (12.8%, *P* = 0.098) in the liver of piglets. Regardless of BW, piglets receiving NT diet had significantly increased the mRNA expressions of GR (by 43.0%), CuZn-SOD (by 29.3%) and Nrf2 (by 31.1%) in the liver relative to piglets receiving CON diet (*P* < 0.05), while significantly increased the mRNA expressions of Keap1 (by 31.7%) in the liver (*P* < 0.05). In addition, the mRNA expression of GP_X_ (by 16.0%) tended to increase in the liver of piglets receiving NT diet (*P* = 0.084).

**Table 3:** Effect of dietary nucleotides supplementation on antioxidant-related gene expressions in the liver of intrauterine growth retarded (IUGR) and normal birth weight (NBW) neonates

Items	CON		NT		SEM	*P* values		
	NBW	IUGR	NBW	IUGR		BW	Diet	BW × diet
CAT	1.00	1.02	1.06	1.21	0.05	0.645	0.498	0.747
iNOS	1.00	0.92	0.86	1.26	0.08	0.278	0.496	0.122
GP_X_	1.00	0.75	1.07	0.96	0.04	0.067	0.084	0.244
GR	1.00^a,b^	0.72^a^	1.14^a,b^	1.32^b^	0.06	0.725	0.026	0.085
CuZn-SOD	1.00^a,b^	0.74^a^	1.22^b^	1.03^a,b^	0.05	0.034	0.047	0.537
Mn-SOD	1.00	0.97	1.03	1.05	0.03	0.475	0.233	0.562
ESOD	1.00	0.76	0.92	0.67	0.09	0.130	0.479	0.878
Nrf2	1.00^a,b^	0.83^a^	1.26^b^	1.14^b^	0.09	0.098	0.003	0.788
Keap1	1.00^a,b^	1.24^b^	0.74^a^	0.79^a^	0.14	0.286	0.021	0.609

### Antioxidant-related genes in the jejunum

The mRNA levels of antioxidant-related parameters in the jejunum after dietary NT supplementation are listed in Table 4. IUGR piglets had a lower mRNA expression of GP_X_ (by 22.5%) and ESOD (by 22.0%) in the jejunum than that of NBW piglets (*P* < 0.05). Irrespective of BW, piglets receiving NT diet had significantly increased the mRNA expression of GP_X_ (by 22.7%), CuZn-SOD (by 39.7%) and ESOD (by 31.0%) in the jejunum relative to piglets receiving CON diet (*P* < 0.05). Additionally, in comparison with piglets receiving CON diet, the mRNA expression of CAT (by 21.3%, *P* = 0.099) and Nrf2 (by 16.5%, *P* = 0.071) tended to increase in the jejunum of piglets receiving NT diet.

**Table 4:** Effect of dietary nucleotides supplementation on antioxidant-related gene expressions in the jejunum of intrauterine growth retarded (IUGR) and normal birth weight (NBW) neonates

Items	CON		NT		SEM	*P* values		
	NBW	IUGR	NBW	IUGR		BW	Diet	BW × diet
CAT	1.00	0.88	1.08	1.20	0.11	0.997	0.099	0.324
iNOS	1.00	0.78	0.91	0.84	0.14	0.529	0.638	0.365
GP_X_	1.00^a^	0.81^a^	1.27^b^	0.95^a^	0.07	0.004	0.014	0.398
GR	1.00	0.91	1.09	1.20	0.13	0.940	0.176	0.476
CuZn-SOD	1.00^a^	0.89^a^	1.41^b^	1.23^a,b^	0.13	0.266	0.009	0.786
Mn-SOD	1.00	0.95	1.02	1.19	0.12	0.636	0.343	0.405
ESOD	1.00^a,b^	0.68^a^	1.18^b^	1.02^a,b^	0.11	0.031	0.023	0.636
Nrf2	1.00	0.88	1.14	1.05	0.08	0.201	0.071	0.870
Keap1	1.00	1.28	0.97	1.13	0.13	0.124	0.495	0.659

### Mitochondrial DNA contents in the liver and jejunum

**Fig. 1:**
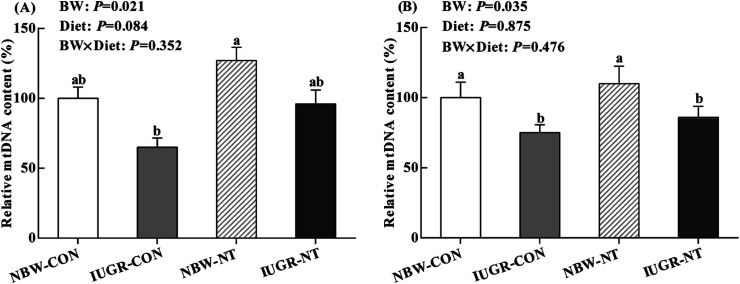
Effects of nucleotides supplementation on hepatic (A) and jejunal (B) mtDNA content of IUGR and NBW neonates (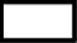, NBW-CON; 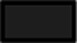, IUGR-CON; 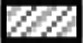, NBW-NT; 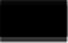, IUGR-NT). CON, control diet; NT, nucleotides diet; BW, body weight; IUGR, intrauterine growth retarded; NBW, normal birth weight; values are means with their standard errors represented by vertical bars (*n* = 7 for each group); columns with different superscript letters mean significantly different between four groups (*P* < 0.05).

IUGR significantly decreased the relative mtDNA content in the liver (Fig. 1A) and jejunum (Fig. 1B) of piglets (by 29% and 23%, respectively, *P* < 0.05). Regardless of BW, piglets receiving NT diet had significantly increased the relative mtDNA content in the liver relative to piglets receiving CON diet (by 35%, *P* < 0.05), but no significantly effect on the relative mtDNA content in the jejunum (*P* > 0.05).

### Mitochondrial DNA biogenesis-related genes in the liver

As shown in Table 5, regardless of diet, IUGR piglets had a lower mRNA expression of PGC-1a in the liver than that of NBW piglets (by 27%, *P* < 0.05). Irrespective of BW, piglets receiving NT diet had significantly increased the mRNA expression of PGC-1a (by 28%, *P* < 0.05) and NRF-1 (by 34%, *P* < 0.05) in the liver relative to piglets receiving CON diet.

**Table 5:** Effect of dietary nucleotides supplementation on mitochondrial biogenesis-related gene expressions in the liver of intrauterine growth retarded (IUGR) and normal birth weight (NBW) neonates

Items	CON		NT		SEM	*P* values		
	NBW	IUGR	NBW	IUGR		BW	Diet	BW × diet
PGC-1α	1.00^a,b^	0.72^b^	1.26^a^	0.94^a,b^	0.12	0.028	0.047	0.469
TFAM	1.00	0.99	1.08	1.18	0.20	0.512	0.216	0.328
NRF-1	1.00^a,b^	0.83^b^	1.17^a^	1.28^a^	0.12	0.218	0.018	0.583
mt SSB	1.00	0.79	0.77	0.93	0.21	0.572	0.643	0.187
mt polr	1.00^a^	0.89	1.01	0.93	0.09	0.685	0.763	0.832
SIRT-1	1.00	0.97	1.21	1.03	0.07	0.437	0.136	0.774

### Mitochondrial DNA biogenesis-related genes in the jejunum

Table 6 presents the mRNA levels of mitochondrial biogenesis-related genes in the jejunum. IUGR had no influence on mitochondrial biogenesis-related genes in the jejunum of piglets (*P* > 0.05). Regardless of BW, piglets receiving NT diet had significantly increased the mRNA expressions of NRF-1 in the jejunum relative to piglets receiving CON diet (by 34%, *P* < 0.05).

**Table 6:** Effect of dietary nucleotides supplementation on mitochondrial biogenesis-related gene expressions in the jejunum of intrauterine growth retarded (IUGR) and normal birth weight (NBW) neonates

Items	CON		NT		SEM	*P* values		
	NBW	IUGR	NBW	IUGR		BW	Diet	BW × diet
PGC-1α	1.00	1.03	1.24	1.21	0.19	0.762	0.167	0.831
TFAM	1.00	1.06	0.97	1.16	0.15	0.459	0.983	0.838
NRF-1	1.00^a^	0.84^b^	1.37^a^	1.10^a^	0.11	0.160	0.036	0.328
mt SSB	1.00	0.86	0.78	0.92	0.12	0.615	0.532	0.413
mt polr	1.00	1.02	1.04	1.00	0.18	0.918	0.875	0.745
SIRT-1	1.00	0.83	0.92	1.11	0.11	0.612	0.424	0.312

## Discussion

It is well established that IUGR followed by accelerated postnatal growth is strongly associated with increased risk of metabolic diseases in later life.^33,34^ Generally, oxidative stress has been shown mainly contributed to metabolic and cardiovascular disease.^35^ Recently, there has been much interest in antioxidant mechanisms which might explain relationships between foods and health outcomes.^36,37^ Nucleotides are conditionally essential nutrients that modulate lipid metabolism, immune function and have a reparative effect in pathological conditions that demand intense nucleic acid and protein synthesis, such as intensive growth, maintaining some organ functions, and repair of certain tissues.^11,38,39^ However, it is unknown whether dietary NT supplementation during the early postnatal period can affect the antioxidant status of piglets with IUGR. The activities of both enzymatic (CAT, CuZn-SOD, T-SOD and GP_X_) and non-enzymatic (GSH, GSSG and T-AOC) antioxidants, MDA content in the plasma and liver, and mtDNA content in the liver and jejunum, as well as gene expression of antioxidant and mitochondrial biogenesis-related signaling molecules in the liver and jejunum were determined in the current study.

### Effects of IUGR and nucleotides supplementation on the antioxidant parameters of the plasma and liver

Oxidative stress can lead to overproduction of ROS and cause lipid peroxidation in organisms.^40^ MDA is one of the toxic metabolites produced by lipid peroxidation,^41^ which is a widely used marker of lipid peroxidation caused by ROS.^42^ Previous study has shown that a marked ROS accumulation was observed in neonates with IUGR.^8^ Since ROS induced lipid peroxidation is one of the important causes of cell damage, the content of MDA was determined. In the present study, we found that IUGR piglets had a higher plasma MDA concentration than NBW piglets, which shows that lipid peroxidation could be increased by IUGR. In addition, the present study observed IUGR piglets decreased the level of plasma T-AOC compared to NBW piglets, suggesting the endogenous antioxidative capability of neonates with IUGR may be impaired.^43^ Interestingly, dietary NT supplementation significantly improved the antioxidant ability of piglets in the current study, as indicated by the decreased liver MDA concentration and increased plasma T-AOC. Given that previous studies have demonstrated that feeding NT supplemented formula increased body weight gain and enhanced the health status of infants,^11,44^ we speculated that antioxidative activity of NT might be involved in the mechanism.

The antioxidant defense system controls the redox balance, a series of enzymes act as the system including SOD, CAT and GP_X_.^45^ These enzymes are the first line of defense against ROS and are generally referred to as primary antioxidants.^46^ In the present study, the activity of plasma GP_X_ and liver T-SOD tended to decrease in IUGR piglets. It has been established that SOD is generally recognized as one of the main antioxidant enzymes; the superoxide anion is converted to H_2_O_2_ by SOD, which can be converted into oxygen through coupled reactions with the conversion of GSH into GSSG, catalyzed by GP_X_.^40,47^ Therefore, the GSH : GSSG ratio has been shown to be a good measure of oxidative stress of an organism.^48^ Consistent with our previous study,^49^ we found IUGR had a tendency to decrease the GSH : GSSG ratio in the liver of piglets, which indicated that IUGR led to a poor antioxidant defense system. In the present study, however, we found that dietary NT supplementation significantly increased the T-SOD, CuZn-SOD and the GSH : GSSG ratio in the plasma, and increased the activities of T-SOD, CuZn-SOD and GP_X_ in the liver of piglets. Most importantly, IUGR piglets fed with NT diet had a comparable T-SOD activity with NBW piglets with CON diet, and significantly decreased the concentration of MDA in the liver relative to IUGR piglets fed with CON diet. These results suggest that dietary NT supplementation may improve the antioxidative ability of IUGR piglets. The finding of the beneficial effect of NT is in agreement with a rodent report in which dietary NT supplementation extend the life span through improving antioxidative ability of rats, as represented by the increased SOD and GP_X_ concentrations.^18^

### Effects of IUGR and nucleotides supplementation on the antioxidant-related gene expression in the liver and jejunum

The liver, which has high oxygen consumption, acts as a crucial innate physical barrier that is responsible for filtering of potentially destructive antigens before they reach the body.^50^ Antioxidant enzyme defense is critical in reducing oxidative stress and maintaining redox homeostasis within the cell.^51^ Previous study has demonstrated that liver oxidative injury and abnormal expression of antioxidant-related enzymes were observed in neonates with IUGR.^52^ In line with this, we found that mRNA expressions of GP_X_ and CuZn-SOD in IUGR piglets was lower than NBW littermates in the current study. The intestine is not only for digestion and absorption of nutrients,^31^ but also possesses several antioxidant defense systems.^53^ Wang *et al.* reported that the impaired antioxidant response in the early postnatal period is a component of the mechanisms responsible for intestinal dysfunction in IUGR neonates.^53^ In our study, consistently, the mRNA expressions of GP_X_ and ESOD showed a down-regulation in the jejunum of piglets with IUGR. However, dietary NT supplementation markedly improved the mRNA expressions of GR and CuZn-SOD in the liver, and increased the GP_X_, CuZn-SOD and ESOD in the jejunum, which indicated that NT could enhance the antioxidative capability of organs through improving the transcription level of antioxidant-related genes. Previous studies confirmed that Nrf2 is a transcriptional regulator of a cellular antioxidant pathway by activating the gene expression of a group of antioxidant enzymes.^54^ Keap1, as a Nrf2-binding protein, which can negatively regulate Nrf2 activity by facilitating Nrf2 degradation *via* the proteasome and inhibiting Nrf2 nuclear translocation in the cytoplasm.^55,56^ Accordingly, the increased Nrf2 mRNA expression and decreased Keap1 mRNA expression in the liver provide another line of evidence for the enhanced of antioxidant enzymes, which resulted from dietary NT supplementation during the suckling period.

### Effects of IUGR and nucleotides supplementation on the mitochondrial biogenesis in the liver and jejunum

Oxidative stress induced by IUGR was associated with mitochondrial DNA damage.^9,57^ Previous study has shown that mtDNA content was reduced in liver and skeletal muscle of IUGR rats offspring.^58^ Similarly, IUGR significantly decreased hepatic and jejunal mtDNA content of piglets in this study. However, piglets fed NT diet tended to increase mtDNA content in the liver, indicating that NT supplementation might enhance the mitochondrial biogenesis. It has been also reported that dietary NT supplementation had the potency to reduce DNA damage induced by oxidative stress in chicken and piglets.^16,17^ The alteration of mtDNA content is under the control of mitochondrial biogenesis, which needs the interaction of multiple transcriptional factors.^59^ PGC-1α and NRF-1 are major transcriptional coactivators of nuclear receptors to modulate mitochondrial biogenesis.^60^ Consistent with previous study,^52^ mRNA level of hepatic PGC-1α were down-regulated by IUGR in the present study. However, dietary NT supplementation significantly improved the mRNA levels of hepatic PGC-1α and NRF-1 and jejunal NRF-1. In line with this, clinical research has been shown that enhancing NT synthesis pathways can reverse mitochondrial biogenesis.^61^ Taken together, in this study, NT supplementation could prevent the reduction of mtDNA content in IUGR piglets through increasing the expressions of mitochondrial biogenesis-related genes.

## Conclusion

Our results demonstrated that dietary NT supplementation can prevent the negative effect of IUGR on oxidative status and mitochondrial biogenesis in liver and jejunum of piglets during the suckling period. This study provides new insights into the role of NT in enhancing antioxidant capability of neonates with IUGR. Further investigation is required to determine the underlying mechanism of NT supplementation on improving antioxidant capability of infants.

## Conflicts of interest

The authors declare that they have no conflicts of interest.

## Supplementary Material

RA-008-C8RA00701B-s001
