# Supplementary material for: Dietary nucleotides supplementation during the suckling period improves the antioxidative ability of neonates with intrauterine growth retardation when using a pig model
Source: RSC Adv. 2018 Apr 30;8(29):16152–60. doi: 10.1039/c8ra00701b (PMC9080267; doi:10.1039/c8ra00701b)
Supplement: RA-008-C8RA00701B-s001 [file RA-008-C8RA00701B-s001.pdf]

This journal is © The Royal Society of Chemistry 2018

Supplementary table 1. Composition and nutrient level of the basal formula milk powder (87.5 % DM basis, %)

Ingredients	%
Whole-milk powder (24% CP)	58.00
Whey protein concentrate (34% CP)	25.00
Casein	5.70
Coconut oil	10.00
CaH_2PO_4	0.10
Choline chloride (50%)	0.10
Vitamin premix*	0.10
Mineral premix†	0.50
L-Arg (98.5%)	0.06
DL-Met (98.5%)	0.06
L-Lys·HCl (78.5%)	
L-Thr (98%)	0.03
L-Trp (98%)	0.05
Total	100.00
Nutrient content	
Digestible energy (kJ/kg)	18390
CP (%)	25.30
Ca (%)	1.02
Total P (%)	0.81
Available P (%)	0.67
Digestible Lys (%)	1.93
Digestible Met (%)	0.63
Digestible Arg (%)	0.86

CP, crude protein.

*Vitamin premix provided per kg powder diet: vitamin A, 0.94 mg; vitamin D_3, 0.01 mg; vitamin E, 20 mg; vitamin K_3, 1 mg; vitamin B_{12}, 0.04 mg; riboflavin, 5 mg; niacin, 20 mg; pantothenic acid, 15 mg; folic acid, 1.5 mg; thiamin, 1.5 mg; pyridoxine, 2 mg; biotin, 0.1 mg.

†Mineral premix provided per kg powder diet: Zn, 90 mg; Mn, 4.0 mg; Fe, 90 mg; Cu, 6.0 mg; I, 0.2 mg; Se, 0.3 mg.

Supplementary table 2. Primer sequences of target and reference genes

Gene	Genebank accession No.	Primer sequence	Amplification length (bp)	Annealing temperature (°C)
CAT	NM_214301.2	5'- ACTTCTGGAGCCTACGTCCT-3'<br>5'-ATCCGTTCATGTGCCTGTGT-3'	93	60
iNOS	NM_001143690.1	5'-AGAGCCTCTGGACCTCAACA-3'<br>5'-CTCACAGCAGAGTTCCACCA-3'	136	60
GP_X	NM_214201.1	5'-GCTCGGTGTATGCCTTCTCT-3'<br>5'-AGCGACGCTACGTTCTCAAT-3'	103	60
GR	AY368271.1	5'-CTACGTGAGCCGACTGAACA-3'<br>5'-TCAGGATGTGAGGAGCTGTG-3'	146	60
CuZn-SOD	GU944822.1	5'-GAGACCTGGGCAATGTGACT-3'<br>5'-CTGCCCAAGTCATCTGGTTT-3'	139	60
Mn-SOD	NM_214127.2	5'-TGGAGGCCACATCAATCATA-3'<br>5'-AGCGGTCAACTTCTCCTTGA-3'	136	60
ESOD	NM_001078688.1	5'-ACGCTGCTCTGTGCTTACCT-3'<br>5'-TCAACTCCTGCCAGATCTCC-3'	142	60
Nrf2	XM_003133500.5	5'-GCCCCTGGAAGCGTTAAAC-3'<br>5'-GGACTGTATCCCCAGAAGGTTGT-3'	67	60
Keap-1	NM_001114671.1	5'-ACGACGTGGAGACAGAAACGT-3'<br>5'-GCTTCGCCGATGCTTCA-3'	56	60
PGC-1a	NM_213963	5'-CCCGAAACAGTAGCAGAGACAAG-3'<br>5'-CTGGGGTCAGAGGAAGAGATAAAG-3'	111	60
TFAM	AY923074.1	5'-GGTCCATCACAGGTAAAGCTGAA-3'<br>5'-ATAAGATCGTTTCGCCCAACTTC-3'	167	60
NRF-1	AK237171.1	5'-GCCAGTGAGATGAAGAGAAACG-3'<br>5'-CTACAGCAGGGACCAAAGTTCAC-3'	166	60
mt SSB	AK352341.1	5'-CTTTGAGGTAGTGCTGTGTCG-3'<br>5'-CTCACCCCTGACGATGAAGAC-3'	143	60
mt polr	XM_001927064.1	5'-CTTTGAGGTTTTCCAGCAGCAG-3'<br>5'-GCTCCCAGTTTTGGTTGACAG-3'	119	60
SIRT-1	EU030283.2	5'-TGACTGTGAAGCTGTACGAGGAG-3'<br>5'-TGGCTCTATGAAACTGCTCTGG-3'	143	60
β-actin	DQ845171.1	5'-GGCGCCCAGCACGAT-3'<br>5'-CCGATCCACACGGAGTACTTG-3'	66	60
GAPDH	NM_001206359.1	5'-TCGGAGTGAACGGATTTGGC-3'<br>5'-TGCCGTGGGTGGAATCATAC-3'	147	60

CAT, catalase; iNOS, nitric oxide synthase 2, inducible; GP_X, glutathione peroxidase; GR, glutathione reductase; CuZn-SOD, copper/zinc superoxide dismutase, cytoplasmic; Mn-SOD, manganese superoxide dismutase, mitochondrial; ESOD, extracellular superoxide dismutase; Nrf2, nuclear erythroid 2-related factor 2; Keap1, Kelch-like ECH-associated protein 1; GAPDH, glyceraldehyde-3-phosphate dehydrogenase; PGC-1α, PPARγ coactivator-1α; TFAM, mitochondrial transcription factor A; NRF-1, nuclear respiratory factor-1; mt SSB, mitochondrial single-strand DNA-binding protein; mt polr, mitochondrial polymerase r; SIRT-1, mammalian silencing information regulator.

Supplementary table 3. Primer and probe sequences used for determination of mtDNA content.

Gene	Genebank accession No.	Primer sequence	Amplification length (bp)	Annealing temperature (°C)
Mitochondrial D-loop	AF276923	5’-GATCGTACATAGCACATATCATGTC-3’<br>5’-GGTCCTGAAGTAAGAACCAGATG-3’<br>5’- (FAM) CCAGTCAACATGCGTATCACCACCA(Eclipse) -3’	198	60
β-actin	DQ452569	5’-CCCCTCCTCTCTTGCCTCTC -3’<br>5’-AAAAGTCCTAGGAAAATGGCAGAAG -3’<br>5’- (FAM) TGCCACGCCCTTTCTCACTTGTTCT (Eclipse) -3’	74	60
